# Screen time and myopia: A serial multiple mediator SEM analysis

**DOI:** 10.3389/fpubh.2022.860098

**Published:** 2022-10-10

**Authors:** Jinchen Xie, Chuntian Lu, Jie Zhu

**Affiliations:** Department of Sociology, Xi'an Jiaotong University, Xi'an, China

**Keywords:** myopia, screen time, sedentary behavior, physical activity, college students

## Abstract

**Background:**

COVID-19 has influenced education systems worldwide, and significantly increased screen time for college students, posing a potential risk of myopia. In China, ninety percent of college students suffer from myopia. Excessive screen time changes college students' lifestyles, imposes potential health risks, and affects opportunities for employment. It is important to identify the potential correlation between screen time use and myopia among college students.

**Methods:**

This paper conducted a nationwide experiment using Chinese college students and set a multiple-mediator SEM model to analyze the potential correlation between screen time and myopia. The two mediators were sedentary behavior and physical activity, respectively.

**Results:**

We obtained three valuable conclusions as follows: First, there was no significant direct relationship between screen time and myopia among Chinese college students during the COVID-19 pandemic. Second, sedentary behavior and physical activity significantly predicted the increase/decrease of myopia among Chinese college students, respectively. Third, a serial multiple mediator that encompassed sedentary behavior and physical activity sequentially fully mediated the relationship between screen time and myopia.

**Conclusions:**

Although there was no directly significant relationship between screen time and myopia, screen time can indirectly influence the risk of suffering myopia by influencing sedentary behavior and physical activity. Our study demonstrates the need to prevent the potential influence of overuse of electronic devices on myopia in college students, especially during the COVID-19 pandemic.

## Introduction

Myopia has been cited as a critical public health risk for college students during the coronavirus 2019 (COVID-19) pandemic. Data indicates that 90% of schools have elected to close or partially close, affecting nearly 3.7 billion students (1). Myopia is most often caused by abnormal axial length growth of the eye resulting in a longer eye with light focused in front of the retina, which causes uncorrected refractive errors, myopic macular degeneration, and even blindness (2). Meanwhile, the cost of myopia in college students is a tremendous financial burden for nearly all countries in the world (3). In Singapore, the direct cost of treating myopia is estimated at US $755 million annually, and globally at US $328 billion per year (4). Existing literature confirms that college is a peak period of myopia development (5). Therefore, it is vital to identify the potential environmental risks which cause myopia in college students. This is especially worth noting in China where there is the largest number of college students worldwide (6).

The extant literature did not provide a consistent relationship between screen time (ST) and myopia. On the one hand, several empirical research studies suggest that the increase of ST significantly predicted the prevalence of myopia (7, 8). Researchers from China, the United States, and India revealed the significant positive relation between screen time and myopia in college students (9, 10). Of particular concern is that the relationship between ST and myopia may be nonlinear, meaning that the risk of myopia tends to increase exponentially as ST rises (11), especially during the COVID-19 outbreak (12). On the other hand, some researchers found no significant correlation between ST and college students' myopia. Several East Asian researchers declared that myopia prevalence emerged without the introduction of electronic equipment in some countries (13). An emerging meta-analysis encompassing 49,789 individuals suggested that screen time was not related to myopia (14).

While extant literature focused exclusively on the direct correlation between ST and myopia, it ignored the potential association between these two factors. Such oversimplification might lead to a biased estimation and become a gimmick for advertisers to promote electronic products and ignore the potential risks it poses to college students' vision. In daily life, ST is closely associated with sedentary behavior (SB) and physical activities (PA), and sufficient empirical evidence supports that SB and PA predict the increase/decrease of myopia, respectively. Before the prevalence of digital devices, SB had long been regarded as the primary factor in explaining myopia development. Almost all empirical studies found that SB positively predicted the development of myopia, whether in China, Europe, or India (15–17). Meanwhile, the increase in PA also significantly decreased the development of myopia. A representative cluster-randomized intervention-controlled trial (*n* = 693) in Taiwan revealed that PA significantly reduced the risk of myopia in respondents (18).

It is important to consider the potential relationship between ST and myopia during the COVID-19 epidemic. From a national perspective, many countries required college students to study online during the COVID-19 epidemic. From an individual perspective, individual college students experienced poor mental health during the COVID-19 epidemic and would be more likely to relax by using electronic devices. Meanwhile, SB and PA significantly increased and decreased, respectively, during the COVID-19 epidemic. Accordingly, the potential relationship between ST, SB, PA, and myopia may become more complex, during the COVID-19 epidemic. Therefore, it is important to reveal the potential relationship between ST and myopia, especially taking the possible mediation of SB and PA into consideration. In this paper we seek to fill this gap, using a nationwide experiment in Chinese college students, and setting a multiple mediator SEM model to analyze the potential correlation between ST and myopia.

## Research hypothesis

**Figure 1:**
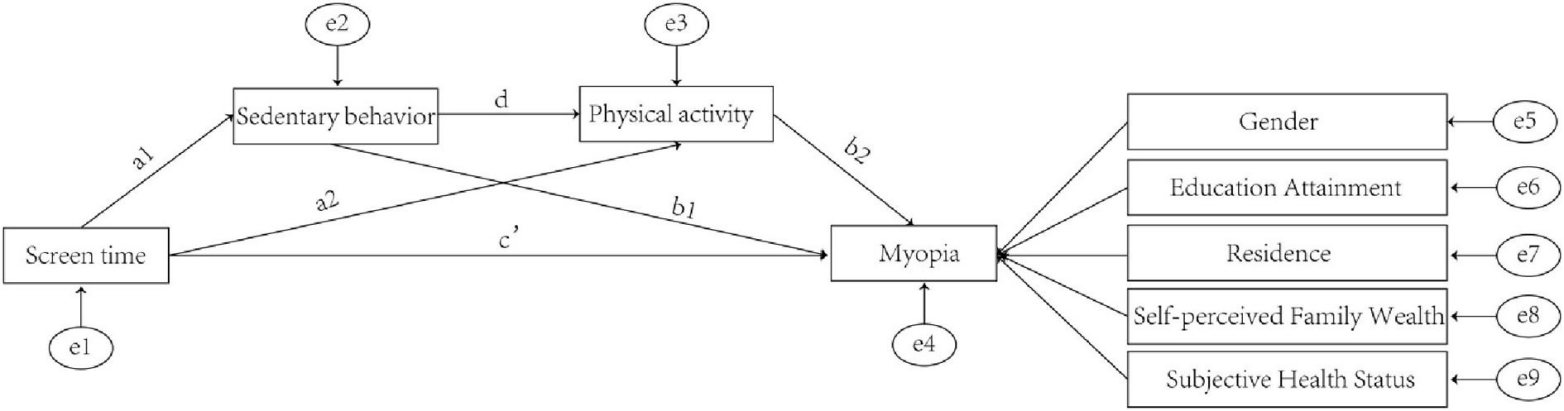
SEM diagram.

In order to clarify the potential association between ST, SB, PA, and myopia, we proposed the following six hypotheses, and the relationship between the four factors is graphically presented in Figure 1.

### The influence of SB on myopia

Realistically, SB is an important factor in the formation of myopia, that is, b1 in Figure 1 is statistically positively significant. SB is conceptualized as any waking behavior characterized by an energy expenditure ≤1.5 metabolic equivalents (METs). For college students who were segregated during the COVID-19 epidemic, SB refers mainly to sedentary reading, and the relationship between sedentary reading and myopia has been consistently reported. The frequency and duration of sedentary reading were significantly and positively associated with the risk ratio of developing myopia in the United States (19), China (20), and Japan (21). Even in Sweden, where schoolwork is very stressful, this influence was positively significant (22). Therefore, we propose the first hypothesis of this study:

*Hypothesis 1, SB positively and significantly predicts the risk of myopia*.

### The influence of PA on myopia

Myopia is a refractive error triggered by prolonged inappropriate indoor eye use, and PA apparently helps alleviate this disadvantage, namely b2 in Figure 1 is statistically negatively significant. During the COVID-19 epidemic, PA was one of the few ways to relieve eye stress among college students. Moderate PA reduced indoor eye time among college students, which was widely demonstrated. It is also worth noting that the potential of PA for myopia relief is enormous (23, 24). A World Health Organization report states that 70% of college students lack sufficient PA, and if college students were motivated to have more PA, it would go a long way toward alleviating the myopia epidemic. We, therefore, propose the second research hypothesis:

*Hypothesis 2, PA negatively and significantly predicts the risk of myopia*.

### The influence of ST on SB

In the context of the COVID-19 epidemic, the relationship between ST and SB is significant, this phenomenon is even more pronounced in China. This is because during the COVID-19 epidemic, China required that almost every college student learn online. Chinese universities, unlike many European universities, require students to complete a vast amount of coursework, and online learning forces Chinese college students to spend more time completing coursework, significantly increasing sedentary time. Therefore, we propose the third research hypothesis:

*Hypothesis 3, ST positively and significantly predicts SB*.

### The influence of ST on PA

From an academic perspective, ST and PA are mutually exclusive, and this relationship has been widely reported, that is, a1 in Figure 1 is statistically positively significant. This phenomenon is even more evident in college students, compared to their elementary and middle school student counterparts. This is because college students are unsupervised in their use of electronic devices, which creates two pathways for ST to influence PA. On the one hand, ST directly reduces the PA time, because for a given college individual, spare time is generally regarded as a constant (25). On the other hand, excessive ST would generate negative emotions and indirectly influence PA (26). Therefore, we propose the fourth research hypothesis:

*Hypothesis 4, ST negatively and significantly predicts PA*.

### The influence of SB on PA

Academically speaking, SB negatively influences PA, similar to the way ST influences PA, namely a2 in Figure 1 is statistically negatively significant. On the one hand, SB directly reduces the amount of time individual college students spend engaging in PA. On the other hand, excessive SB will cause irreversible physical issues such as obesity, varicose veins, and even lumbar disc herniation, influencing college students' physical functions and hindering them from physical activity. Therefore, we propose the fifth research hypothesis:

*Hypothesis 5, SB negatively and significantly predicts PA*.

### The influence of ST on myopia

The relationship between ST and myopia has not been consistently reported. On the one hand, some scholars have suggested a positive relationship between ST and myopia (8, 27). On the other hand, some researchers have demonstrated no statistically significant relationship between ST and myopia within a large data sample (13, 14). Moreover, these arguments exist in many countries, such as Australia, the United States, China, and New Zealand (28).

Thus, we suggest that SB influences myopia through three pathways: (a) ST increases SB and leads to increased myopia, as in a1-b1, (b) ST decreases PA and leads to increased myopia, as in a2-b1, and (c) ST increases SB and leads to decreased PA, and ultimately increased myopia, as in a1-d-b2. Taken together, because the mediation consisting of SB and PA sequentially, completely mediated the influence of ST on myopia, the direct influence of ST on myopia was not significant. We therefore propose the sixth research hypothesis:

*Hypothesis 6, there is no significant direct relationship between ST and myopia, for a serial multiple mediator that encompassed SB and PA completely mediated the direct effects*.

## Materials and methods

### Study participants

Experimental subjects were recruited for a nationwide experiment among Chinese college students during the COVID-19 epidemic, which aimed at estimating the potential psychological and physical changes of Chinese college students due to the COVID-19 epidemic. We strictly followed the multi-stage stratified random cluster sampling principles. First, we randomly selected seven universities in mainland China. Second, we randomly selected different classes in all universities. Third, we distributed paper questionnaires to selected students, and assigned special personnel to supervise the completion process of questionnaires. Data was collected from 13 Sep to 12 Dec, 2021. Finishing a questionnaire took about 20 min for college individuals. The inclusion standards of valid respondents were as follows: The information of respondents' demographic characteristics, myopia, ST, SB, and PA were validated, simultaneously. After excluding the invalid respondents, the final data size was 759. Our research was authorized by the Institutional Review Board of Xian Jiaotong University and obeyed the ethics code of the World Medical Association Declaration of Helsinki.

### Determination of screen time

The main independent variable in our empirical research was ST. We designed a fill-in-the-blank question to ask respondents how many minutes they used their phones last week as follows:

Q1: During the last week, how many minutes were spent on the phone every day?

In order to ensure the authenticity of the data, individuals were asked to look for the “healthy use of mobile phone” option (Android system) or the “screen use time” option (Apple System) in the “Settings” option of the mobile phone and report it in the questionnaire after converting it into minutes.

### Determination of sedentary behavior and physical activities

The information of SB of respondents was collected by the following question in our research:

Q1: How many minutes did you sit every day last week?
ST was estimated by the continuous variable acquired from the question above.

PA was obtained by the International Physical Activity Questionnaire (IPAQ), which has proven to be a standard questionnaire that effectively displayed the intensity of physical activity of the respondents. The days, hours, and minutes of vigorous, moderate, and mild exercise every day last week were systematically investigated, respectively. The index obtained from the three activities above were multiplied by 8.0, 4.0, and 3.3, respectively, and finally generated the metabolic equivalent (MET) as follows:


(1) $$\begin{array}{l} Met_{total}\;=\;Minutes_{vigorous}*8.0+Minutes_{\mathrm{mod}erate}*4.0\\ \;+\;Minutes_{mild}*3.3 \end{array}$$

Minutes_vigorous_, Minutes_moderate_, Minutes_mild_ denoted the minutes of vigorous, moderate, and mild physical activity performed in the last week. After taking the required number of days as the constraint, A three category variable was obtained, 1, 2, and 3 denote low, mid, and high PA, respectively.

### Determination of myopia

The dependent variable in our research was myopia, a self-administered question was used to investigate the myopia of respondents as follows:

Q1: Which of the following options best indicates your myopia?
A separate four-category option was offered to each respondent.
(a) No myopia, i.e., respondents with 0–50 diopters, were defined as ophthalmologically emmetropia, meaning that the respondent's visual acuity required a Spherical Equivalent Refraction (SER) correction of 0–0.5 for ideal correction.
(b) Mild myopia, i.e., the respondent's spectacles are 50–300 diopters, ophthalmologically meaning that the respondent's vision needs 0.5–3.0 SER correction for ideal correction.
(c) Moderate myopia, i.e., the respondent's spectacles are 300–600 diopters, and ophthalmologically the respondent's visual acuity requires a SER of 3.0–6.0 for ideal correction.
(d) Severe myopia, i.e., the respondent's spectacles are 600 diopters or more, and ophthalmologically the respondent's visual acuity requires an SER correction of 6.0 or more for ideal correction.

### Covariates

Covariates were set to control the statistical bias in the estimation, contained respondents' gender (1, 2 denoted male or female, respectively), education attainment (1, 2, and 3 represented Freshman or Sophomore, Junior or Senior, Master or Doctor, respectively), self-perceived family wealth (1, 2, and 3 denoted low, medium, high, respectively), residence (1, 2 denoted urban and rural, respectively), subjective health status (1, 2, and 3 denoted low, medium, high, respectively). The above factors have been proven to have a significant influence on myopia (29, 30).

### Statistical analysis

We chose to use *MPLUS 8.2* to analyze the potential relationship between ST and myopia in Chinese college students during the COVID-19 epidemic. A series of SEM models were built for this study based on the framework of a serial multiple mediator models that includes multiple mediators that are sequentially linked within a single model (31). This method was appropriate for our research because two moderations were sequentially placed in the association between ST and myopia. Heterogeneous information of respondents was shown in descriptive statistics by the myopia group, to reveal the potential relationship between ST, SB, PA, and myopia preliminarily.

Following the extant literature, we hypothesized that SB and PA indirectly participated in the relationship between ST and myopia. In order to prove the hypothesis, we first verified the relationship between the above four factors through a series of regression models, to provide empirical support for subsequent SEM analysis.

And then, an SEM was designed to describe the potential relationship between ST and myopia, with a serial multiple mediator of SB and PA, sequentially. A modification index (MI) was used to optimize our model through the specific code of *modindices* in MPLUS. Direct and indirect effects of the correlation between ST and myopia were estimated simultaneously. As for the multivariate non-normality, bootstraps (5,000 times) was the repeated sampling technology used in our research to reduce bias. Meanwhile, we took all covariates into consideration.

## Results

### Data describing

Table 1 briefly showed the results of the descriptive statistics. The percentage of college students with myopia accounted for 82.87% of the total students, and mild, moderate, and severe myopia in students consisted of 17.13, 27.01, and 11.33%, respectively. Meanwhile, the proportion of students without myopia in the full sample was only 17.13%, which indicated that myopia was extremely prevalent among Chinese college students during COVID-19.

**Table 1:** Data description and correlation test for all variables.

	**Full sample**	**Myopic Symptoms subgroups**				
		**Non** **(*N* = 130)**	**Slight** **(*N* = 205)**	**Moderate** **(*N* = 338)**	**Severe** **(*N* = 86)**	***P***
Screen time	352.001 (5.809)	341.361 (14.603)	346.512 (9.537)	348.221 (9.065)	396.023 (18.434)	0.057
Sedentary behavior	376.565 (5.584)	331.907 (13.229)	367.731 (10.222)	382.766 (8.289)	440.755 (17.217)	0.000***
**Physical activity**						
Low physical activity	22 (2.90%)	2 (9.09%)	4 (18.18%)	10 (45.45%)	6 (27.27%)	0.000***
Mid physical activity	344 (45.32%)	43 (12.50%)	83 (24.13%)	162 (47.09%)	56 (16.28%)	
High physical activity	393 (51.78%)	85 (21.63%)	118 (30.03%)	166 (42.24%)	24 (6.11%)	
**Gender**						
Female	370 (48.75%)	75 (57.69%)	101 (49.27%)	153 (45.27%)	41 (47.67%)	0.118
Male	389 (51.25%)	55 (42.31%)	104 (50.73%)	185 (52.33%)	45 (51.23%)	
**Grade**						
Freshman or sophomore	656 (86.43%)	119 (91.54%)	117 (86.34%)	288 (85.21%)	72 (83.72%)	0.456
Junior or senior	48 (6.32%)	6 (4.62%)	10 (4.88%)	25 (7.40%)	7 (8.14%)	
Master or doctor	55 (7.25%)	5 (3.85%)	18 (8.78%)	25 (7.40%)	7 (8.14%)	
**Residence**						
Rural	420 (55.34%)	80 (61.54%)	113 (55.12%)	188 (55.62%)	39 (45.35%)	0.138
Urban	339 (44.66%)	50 (38.46%)	92 (44.88%)	150 (44.38%)	47 (54.65%)	
**Self-perceived family wealth**						
Low	29 (3.82%)	9 (6.92%)	7 (3.41%)	9 (2.66%)	4 (4.65%)	0.294
Medium	352 (46.38%)	53 (40.77%)	91 (44.39%)	168 (49.70%)	40 (46.51%)	
High	378 (49.80%)	68 (52.31%)	107 (52.20%)	161 (47.63%)	42 (48.84%)	
**Subjective health status**						
Bad	452 (59.55%)	95 (73.08%)	122 (59.51%)	194 (57.40%)	41 (47.67%)	0.009**
Medium	252 (33.20%)	31 (23.85%)	69 (33.66%)	117 (34.62%)	35 (40.70%)	
Good	55 (7.25%)	4 (3.08%)	14 (6.83%)	27 (7.99%)	10 (11.63%)	
**Correlation test**	**1**	**2**	**3**	**4**	**5**	**6**	**7**	**8**	**9**
1.Screen time	(–)								
2.Myopia	0.088	(–)							
3.Sedentary time	0.002**	0.000***	(–)						
4.Physical activity	0.371	0.000***	0.001***	(–)					
5.Gender	0.379	0.043*	0.000***	0.000***	(–)				
6.Education attainment	0.975	0.115	0.414	0.057	0.118	(–)			
8.Residence	0.267	0.050*	0.000***	0.028*	0.443	0.330	(–)		
7.Self-perceived family wealth	0.111	0.687	0.938	0.155	0.000***	0.595	0.000***	(–)	
9.Subjective health status	0.071	0.000***	0.000***	0.000***	0.689	0.015*	0.334	0.047*	(–)

*Significant at 10%, **significant at 5%, ***significant at 1%.

**Figure 2:**
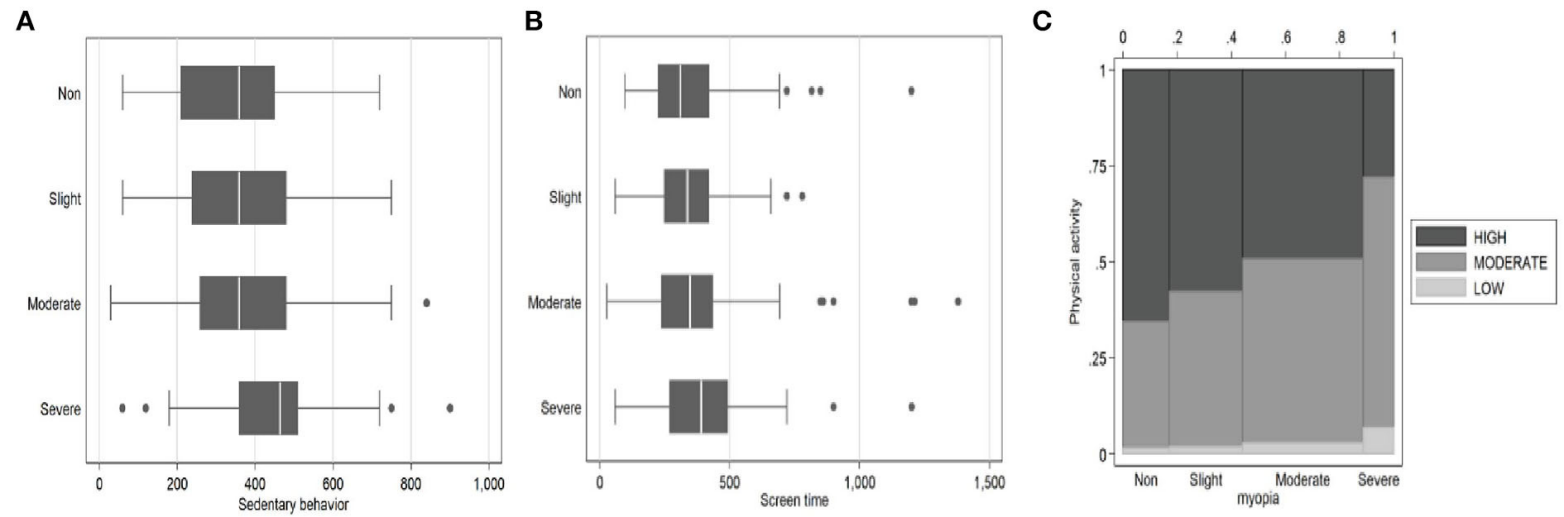
The relation between screen time, sedentary behavior, physical activity, and myopia. **(A)** SB and myopia. **(B)** ST and myopia. **(C)** PA and myopia.

Additionally, Table 1 revealed the statistically significant differences in ST, SB, and PA among individuals with different levels of myopia. To further reveal the association between SB, ST, PA, and myopia, we graphically depicted the relationships between SB and myopia, ST and myopia, and PA and myopia as shown in Figure 2 in (A), (B), and (C), respectively. In the context of descriptive statistics, Figure 2A verifies that high SB predicts high myopia, Figure 2B verifies that nearly no significant relationship between ST and myopia, and Figure 2C verifies that high PA predicts low myopia. In addition, we found some outliers in Figures 2A,B, that support our use of the “Winsor” command for removing the outliers.

As shown in the correlation results, SB, PA, and several covariates were tightly associated with myopia, while there was no statistically significant correlation between ST and myopia. Warranting particular attention, there was a statistically positive significant relationship between ST and SB, and a statistically negative relationship between ST and PA. The results of the correlation analysis preliminarily indicated that a serial multiple mediator that encompassed ST and PA sequentially, might mediate the relationship between ST and myopia.

### Regression analysis

We set up three regression models to further verify the relationship between ST, SB, PA, and myopia. It was worth noting the several outliers in the sample (as shown in Figure 2), therefore we used the “*winsor 2*” command in *stata* to shrink the tails of all regressions by 10%. Model one focused on the relationship between ST and SB, holding all else equal, an increase of 10% in ST was associated with a 1.2% increase in SB, statistically significant (Coefficient = 0.120, SD = 0.037, *P* < 0.05). This conclusion is consistent with the extant literature, especially in the East Asian countries (32, 33). Of interest, these countries were defined characteristically by a high prevalence of online learning; accordingly, online learning might bring an inevitable disadvantage and impair the eyesight of college students.

As shown in the middle two columns of Table 2 (Model 2), a negative correlation between PA and SB could be found. Holding all else equal, an increase of 10% in SB was associated with a 4.9% decrease in PA—statistically significant (Coefficient = −0.491, SD = 0.207, *P* < 0.05)—while the correlation between ST and PA was statistically insignificant (Coefficient = −0.116, SD = 0.208, *P* > 0.05). These conclusions are rarely found in previous literature because the previous studies rarely took ST and SB into the relationship between ST and myopia.

**Table 2:** Regression model results.

	**Model 1:**		**Model 2:**		**Model 3:**	
	**y** = **Sedentary behavior**		**y** = **Physical activity**		**y** = **Myopia**	
	**Coefficient**	**SD**	**Coefficient**	**SD**	**Coefficient**	**SD**
**Physical activity**						
Low PA	(–)		(–)		1	
Mid PA	(–)		(–)		−0.521	0.447
High PA	(–)		(–)		−1.05***	0.45
Sedentary behavior	(–)	−0.491***	0.207	0.573***	0.182
Screen time	0.120***	0.037	−0.116	0.208	0.172	0.185
**Gender**						
Male	1		1		1	
Female	0.089***	0.028	−0.851***	0.158	0.046	0.142
**Grade**						
Freshman and sophomore	1		1		1	
Junior and senior	0.013	0.056	−0.494	0.321	0.388	0.288
Master and doctor	0.043	0.053	−0.443	0.296	0.224	0.259
**Residence**						
Rural	1		1		1	
Urban	0.127***	0.029	−0.212	0.165	0.134	0.147
**Self-perceived family wealth**						
Low	1		1		1	
Medium	0.002	0.029	−0.022	0.166	0.08	0.147
High	−0.192***	0.074	−0.365	0.42	−0.437	0.385
**Subjective health status**						
Bad	1		1		1	
Medium	−0.124***	0.056	0.51	0.309	−0.269	0.283
Good	−0.209***	0.054	1.542***	0.304	−0.486	0.279

^*^Significant at 10%, ^**^significant at 5%, ^***^significant at 1%.

Model 3 reveals the correlation between ST, SB, PA, and myopia, simultaneously. First, the correlation between PA and myopia was statistically significant (Coefficient = 1.05, SD = 0.45, *P* < 0.05). Holding all else equal, college students with high PA had a 5.1% lower relative risk of developing myopia compared with those with low PA. Similar conclusions had been found in Denmark, Australia, and other countries (34, 35). Second, the correlation between SB and myopia was statistically significant and positive (Coefficient = 0.573, SD = 0.182, *p* < 0.05). When all else is constant, an increase of 10% in SB was clearly related to a 5.32% increase in myopia. The conclusion corroborated the findings in the Pearson correlation test. Third, the results indicated no significant association between myopia and ST (Coefficient = 0.172, SD = 0.183, *p* > 0.05). We reached the opposite conclusion compared with a representative meta-analysis which indicated that ST significantly predicted an increased risk of myopia (28). A possible explanation was that the confounding influence of ST and PA was not considered in this meta-analysis, we isolated the net influence of ST by controlling the SB and PA and eventually drew a more accurate result.

### Serial multiple mediator SEM analysis

**Figure 3:**
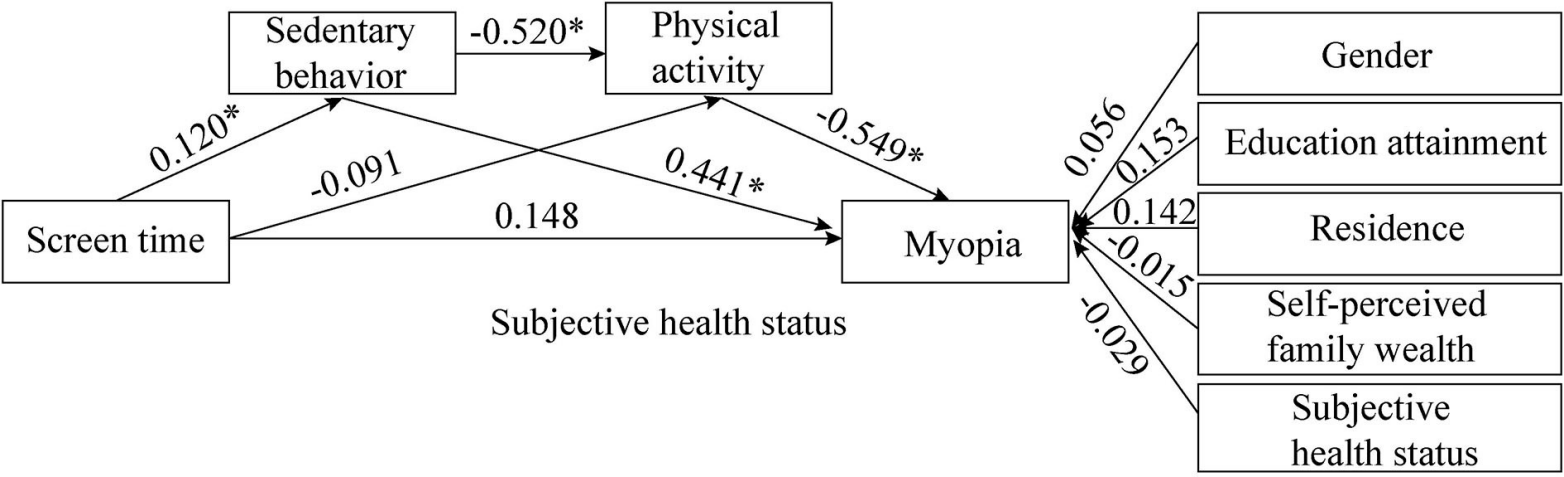
The potential relationship between ST and Myopia (*X*^2^ = 135.289, *p* < 0.05; CFI = 0.944 > 0.90; TLI = 0.971 > 0.90; SRMR = 0.059 < 0.08), *significant at *p* < 0.001.

We set up an SEM to reveal the potential association between ST and myopia. According to Figure 3, the serial multiple mediator model showed acceptable fit statistics: *X*^2^ = 135.289, *p* < 0.05, CFI = 0.944 > 0.90; TLI = 0.971 > 0.90, SRMR = 0.059 < 0.08.

As shown in Table 3, ST was positively associated with SB, statistically significant (Coefficient = 0.120, 95% CI = 0.036; 0.204, *p* < 0.05); thus, hypothesis 3 was verified. While there was no statistically significant relationship between ST and myopia (Coefficient = 0.148, 95% CI = 0.135; 0.432, *p* > 0.05), ST and PA (Coefficient = −0.091, 95% CI = −0.387; 0.204, *p* > 0.05), simultaneously. Meanwhile, a 10% increase in SB and PA was significantly associated with a 4.41% increase (Coefficient = 0.441, 95% CI = 0.158; 0.725, *p* < 0.05) and 5.49% decrease (Coefficient = −0.549, 95% CI = −0.829; −0.268, *p* < 0.05) in myopia, respectively; thus, hypothesis 1 and hypothesis 2 were simultaneously verified. It is worth noting that there was a significant negative relationship between SB and PA (Coefficient = −0.520, 95% CI = −0.855; −0.186, *p* < 0.05); so, hypothesis 5 was verified. Additionally, there is no significant difference in myopia between men and women (Coefficient = 0.056, 95% CI = −0.234;0.346, *p* > 0.05). Similarly, there were no significant differences in myopia among college students between different grades (Coefficient = 0.153, 95% CI = −0.077; 0.383, *p* > 0.05), different residence (Coefficient = 0.142, 95% CI = −0.149; 0.432, *p* >0.05), and different self-perceived family wealth (Coefficient = −0.015, 95% CI = −0.287; 0.258, *p* > 0.05). However, the better the subjective health status, the lower the probability of getting myopia (Coefficient = −0.229, 95% CI = −0.457; −0.002, *p* < 0.05).

**Table 3:** Coefficients and significance tests for the explanatory model pathways.

**Effects**	**Initial model**		***P*-value**
	**Coefficient**	**95%CI**	
(a1) Screen time → Sedentary time	0.120***	0.036; 0.204	< 0.005
(a2) Screen time → Physical activity	−0.091	−0.387; 0.204	>0.005
(b1) Sedentary time → Myopia	0.441***	0.158; 0.725	< 0.005
(b1) Physical activity → Myopia	−0.549***	−0.829; −0.268	< 0.005
(d) Sedentary time → Physical activity	−0.520***	−0.855; −0.186	< 0.005
(c') Screen time → Myopia	0.148	−0.135; 0.432	>0.005
Gender → Myopia	0.056	−0.234; 0.346	>0.005
Grade → Myopia	0.153	−0.077; 0.383	>0.005
Residence → Myopia	0.142	−0.149; 0.432	>0.005
Self-perceived family wealth → Myopia	−0.015	−0.287; 0.258	>0.005
Subjective health status → Myopia	−0.229***	−0.457; −0.002	< 0.005
**Effects of screen time on myopia**			
Total	0.286	−0.065; 0.636	>0.005
Ind.Total	0.137***	0.042; 0.317	< 0.005
Ind1(a1 × b1)	0.053***	0.003; 0.103	< 0.005
Ind2(a2 × b2)	0.050	−0.114; 0.214	>0.005
Ind3(a1 × d × b2)	0.034***	0.004; 0.065	< 0.005

^*^Significant at 10%, ^**^significant at 5%, ^***^significant at 1%.

The last five rows of Table 2 showed the indirect effects including specific and total indirect effects (the combination of specific indirect effects), and total effects (the combination of indirect and total direct effects) in addition to the direct effects. The total effects of the model were 0.286 and statistically insignificant (Coefficient = 0.286, 95% CI = −0.065; 0.636, *p* > 0.05). This is acceptable in our model because there were two opposite-direction mediating paths in the relation between ST and myopia, simultaneously. Additionally, 51.75% of the effects originated through the direct effects between ST and myopia, statistically insignificant (Coefficient = 0.148, 95% CI = −0.135; 0.432), which indicated that there was no direct correlation between ST and myopia. However, the total indirect effects were statistically significant (Coefficient = 0.137, 0.042; 0.317, *p* < 0.05). On the one hand, specific indirect effects through SB accounted for 38.6% of the indirect effect (Coefficient = 0.053, 95% CI = 0.003;0.103, *p* < 0.05). On the other hand, the serial multiple mediator consisting of SB and PA, showed significantly positive indirect effects (Coefficient = 0.034, 95% CI = 0.004–0.065, *p* < 0.05). Eventually, hypothesis 6 was verified.

This model supported that serial mediation, including SB and PA sequentially, existed in predicting the relationship between ST and myopia. Since the coefficients indicated opposing directions, our results revealed a suppressive and promoted the role of SB and PA, respectively. More specifically, an increase of 10% in ST was associated with a 2.86% increase in myopia, though fully mediated by SB and PA. Copious studies supported that there was no significant relationship between ST and myopia, because myopia prevalence increased before the massive use of digital devices in some countries and territories (36). However, we found that a serial multiple mediator consisting of SB and PA, completely mediated the relationship between ST and myopia. In other words, the rise in ST increases SB and reduces PA, and accordingly indirectly increases the risk of myopia.

## Discussion

We obtained three valuable conclusions as follows: First, there was no significant direct relationship between ST and myopia among Chinese college students during the COVID-19 epidemic. Second, SB and PA significantly predicted the increase/decrease of myopia among Chinese college students, respectively. Third, a serial multiple mediator that encompassed SB and PA sequentially, completely mediated the relationship between ST and myopia.

The growth in the use of electronic devices for college students, whether for leisure or information, is common worldwide (37). Meanwhile, this trend is even more pronounced during the prevalence of COVID-19 (38). It is worth noting that previous studies have concluded that myopia occurs mainly in adolescence (age 0–14). And college students are characterized as having a fixed axial length of the eye, thus the change of environmental factors is rarely related to the development of myopia in college students. However, our empirical conclusion confirms that screen time indirectly influenced the development of myopia among college students through the mediation of SB and PA, sequentially. Our findings strongly support a representative study from China which revealed that the overall myopia prevalence increased from 52.89% in June 2019 (before the COVID-19 epidemic) to 53.9% (during the COVID-19 epidemic) in December 2019 and 59.35% in June 2020 (during strict containment of COVID-19) (39). Globally speaking, more than 800 million people are suffering myopia impairment, an empirical estimation suggests that 100 million people experienced moderate-to-severe distance vision impairment or blindness during the COVID-19 epdiemic (40). Electronic devices are considered an important tool to suppress depression and reduce social isolation. Several literatures indicate that college students use digital devices as a tool for stress relief (41). Accordingly, the significant rise in mental stress among college students during the COVID-19 epidemic directly triggered dependence on electronic devices, increased screen time, and indirectly caused irreversible damage to vision.

However, consistent evidence of the association between ST and myopia is lacking. On the contrary, several studies reveal an irrelated correlation between ST and myopia, especially for college students. But all of them overlooked the potential link between the two factors, namely that ST might influence other environmental factors, and finally indirectly influence the development of myopia. If researchers ignore the indirect relationship between ST and myopia, they will arbitrarily believe that there is no direct correlation between these two factors. This misconception is likely to be a gimmick for electronic companies to promote their products as convenient but not harmful to health. Our empirical results reveal the potential relationship between ST and myopia as follows: ST indirectly influences myopia by influencing a serial multiple mediator consisting of SB and PA. That is to say, the rise in ST increases SB and reduces PA, and finally indirectly increases the risk of myopia.

The added value of this study is 3-fold. First, we seek to fill the gap left by previous studies that have neglected the potential correlation between ST and myopia, which is helpful in answering the question of whether increased screen time would increase the risk of myopia. Second, because prior researchers have rarely taken other environmental factors into consideration, we constructed a SEM including the ST, SB, PA, and myopia, simultaneously. This strategy is beneficial to more clearly determine the relationship between the four factors. Third, we focus on college students, who have received little attention in previous myopia studies.

In the context of policy, these results underpin several initiatives supporting the restricted use of digital devices by college students. During the COVD-19 epidemic, college students have been exposed to digital devices for a longer time, and they need to use a digital device not only to complete online learning but also to relieve stress and depression. However, too much screen time will lead to irreversible refractive errors, causing more severe myopia, and finally impaired quality of life. It is gratifying that an increasing number of countries have been focusing on the overuse of electronic devices among college students and are introducing policies to curb the deterioration resulting from this phenomenon. The Chinese government has introduced regulations to limit the time for digital devices among college students (42). To cope with the prevalence of myopia during the epidemic, a policy from the U.S. Federal Government provides guidelines to restrict digital use for college students (43). This research supports that it is vital to strengthen policies to prevent the overuse of digital devices, and to advocate for a healthy lifestyle.

There are some limitations to be interpreted carefully. First, our sample size was not highly sufficient (*N* = 759), which would shape several limitations on the generalization of the study results. Second, several variables were collected by a self-administered questionnaire, which might lead to unintentional estimated bias. Third, given the cross-sectional design of this study, the data do not address causality, but associations instead.

Despite these limitations, our research clearly revealed the potential relationship between ST and myopia. Accordingly, this study is of vital importance both theoretically and empirically, and it provides some meaningful insights for future research work. From a data perspective, more cohort research is required to exclude the influence of confounding factors and isolate the net influence between ST and myopia. From a theory perspective, there are questions which need to be further verified in future studies, such as whether this serial multiple mediator will be moderated by other environmental factors, and whether the direction and magnitude of coefficients show significant heterogeneity in samples with different demographic characteristics.

## Conclusion

This paper revealed a potential relationship between ST and myopia, under the serial multiple mediator by SB and PA, sequentially. We found that although there was no directly significant relationship between ST and myopia, ST can indirectly influence the risk of myopia by influencing SB and PA. Our study refutes studies that suggest that there is no correlation between digital devices use and myopia and fills the gap that previous studies have neglected regarding the potential association between ST and myopia. The COVID-19 epidemic is far from ending, even in China, with among the toughest prevention measures in the world. The recent delta-variant virus puts all college students in five Chinese cities of more than 10 million people in online education. Our study demonstrated the need to prevent the potential overuse of electronic devices to mitigate their influence on myopia in college students, especially during the COVID-19 epidemic.

## Data availability statement

The original contributions presented in the study are included in the article/supplementary material, further inquiries can be directed to the corresponding author/s.

## Ethics statement

The studies involving human participants were reviewed and approved by Ethics Committee of the School of Xi'an Jiaotong University. The patients/participants provided their written informed consent to participate in this study.

## Author contributions

JX and JZ: conceptualization. CL and JX: data collection, writing—original draft, and writing—review and editing. JX: methodology. CL: supervision. All authors have read and agreed to the published version of the manuscript.

## Conflict of interest

The authors declare that the research was conducted in the absence of any commercial or financial relationships that could be construed as a potential conflict of interest.

## Publisher's note

All claims expressed in this article are solely those of the authors and do not necessarily represent those of their affiliated organizations, or those of the publisher, the editors and the reviewers. Any product that may be evaluated in this article, or claim that may be made by its manufacturer, is not guaranteed or endorsed by the publisher.
